# Resistance to deltamethrin by domestic and wild *Triatoma infestans* populations in the municipality of Toro Toro, Potosi, Bolivia

**DOI:** 10.1186/s13071-018-2663-5

**Published:** 2018-02-17

**Authors:** Jorge Espinoza Echeverria, Marinely B. Bustamante Gomez, Grasielle Caldas D Ávila Pessoa, Mirko Rojas Cortez, Antonio Nogales Rodriguez, Liléia Gonçalves Diotaiuti

**Affiliations:** 1 Laboratório de Referência Triatomíneos e Epidemiologia da Doença de Chagas, Centro de Pesquisas René Rachou, FIOCRUZ-M G, Belo Horizonte, Brasil; 2 CEADES Salud y Medio Ambiente/Plataforma Chagas, Cochabamba, Bolivia; 3 Servicio Departamental de Salud - Potosi, Programa Nacional de Chagas, Potosi, Bolivia

**Keywords:** *Triatoma infestans*, Bolivia, Chagas disease, Insecticide resistance, Deltamethrin, Macro- and microgeography

## Abstract

**Background:**

Chemical control with pyrethroid insecticides has been effective in reducing endemic areas of distribution of *Triatoma infestans* in the Southern Cone, as well as Bolivia; this had considerably reduced the infestation of households in a large part of the territory. Nowadays, areas such as the Chaco and the Inter-Andean Valleys are regions where the reach of vector control strategies is becoming limited, and infestations of insecticide-treated households are reported more often. The objective of this study was to determine if the persistence of *T. infestans* stems from changes in the susceptibility of its toxicological profile in four communities in the municipality of Toro Toro, Potosi, Bolivia.

**Methods:**

Susceptibility to deltamethrin of wild and domestic populations of *T. infestans* was evaluated in two stages (16 populations before and 13 populations after spraying) among DUs (structures in the intra- and peridomicile) and wild ecotopes, in four communities. Serial dilutions of deltamethrin in acetone (0.2 μl) were applied topically on standardized first-stage nymphs. Dose-response results were analyzed with the software PoloPlus and the relationships between lethal doses (LD) and resistance ratios (RR_50_) were determined.

**Results:**

Different degrees of RR_50_ were detected among the populations before and after spraying (25.66–54.70 and 21.91–40.67, respectively), as well as in different ecotopes within a DU (DU JC 3, 28.06–36.13, in mixed structures of corrals and chicken coops; and DU JG 3, 46.27–25.70, in kitchen roofs), or in the wild environment of the community JG Sil (29.21–40.67). The mortality of insects undergoing diagnostic dose (DD) was never higher than 34%.

**Conclusion:**

The results obtained in this study showed resistance of *T. infestans* to deltamethrin in four communities, hence the complexity of this phenomenon is not only limited to the level of communities, but also applies to the microgeographical level, as in different ecotopes present within the DUs. This phenomenon should be considered while planning the activities of control programs.

## Background

Chagas disease is an important parasitic infection in Latin America, caused by the parasite *Trypanosoma cruzi*, mainly transmitted to humans and mammals by blood-sucking triatomine insects [1, 2]. *Triatoma infestans* Klug (Hemiptera: Reduviidae) is widely distributed in South America [3] and is the main vector species within the endemic area of Bolivia [4–6]. Insecticides play a central role in controlling major vectors of diseases [7]; control programs in Southern Cone countries focus on the interruption of human *T. cruzi* transmission by *T. infestans*, with the application of residual insecticides [8, 9], mainly pyrethroids (particularly deltamethrin) [10–12], because of their efficacy, persistence and low environmental impact [1, 13]. But, chemical control was only partially successful in eliminating domestic triatomine infestation.

Resistance to pyrethroids in triatomines has been detected in South America since the 90s [14–16]. High levels of resistance to pyrethroids, detected in *T. infestans* in certain areas of Argentina and Bolivia, were attributed to possible failures of control programs [17–21]. In the Chaco region, the success of insecticide control campaigns in rural communities is limited by early reinfestation, apparently originating from residual peridomestic foci [5, 22]. Peridomestic sites are the first to be recolonized, sustain dense populations of *T. infestans*, and increase the risk of domestic reinfestation in rural northern Argentina [22, 23].

The municipality of Toro Toro (Department of Potosi) is situated in the Bolivia Inter-Andean Valleys. Before the beginning of the Chagas programme in this department, high infestation rates of *T. infestans* were present. After approximately 15 years of chemical control, the density of *T. infestans* decreased and achieved a compatible level with the vigilance phase [6]. Nevertheless, *T. infestans* persists, and it was not known if this was due to operational failures or to susceptible alterations.

Considering previous reports on the different levels of *T. infestans* resistance in different geographical areas of Bolivia [18, 20, 24–27], the objective of this study was to determine if the origin of *T. infestans* persistence was due to alterations of susceptibility in four communities of the municipality of Toro Toro. Response to insecticide was measured and compared among the different environments (intra, peridomestic and wild environment).

## Methods

### Study area

The study was carried out in the communities of Taqó Taqó (TQ), Julo Chico (JC), Julo Grande (JG) and Calahuta (CA) of the municipality of Toro Toro (Charcas Province), north of Potosi Department, Bolivia. This area is part of the Bolivian-Tucuman biogeographical province [28, 29]. These communities were historically characterized by high rates of house infestation and high triatomine densities. Since 1995, the infestation has decreased and the area has been under vigilance with irregular control activities using alphacypermethrin. The last insecticide spraying campaign occurred the first quarter of 2013 [30].

In these communities farming is the main form of sustenance, especially guava, lemon and also sweet potato production and animal husbandry (cattle, goats, sheep and poultry). The cultivated areas are restricted and very close to the houses, with a very limited production. Housings are frequently precarious and are built with adobe, stone and the majority of houses have some peridomestic structure to protect the domestic animals (i.e. goat corrals and chicken coops).

A domiciliary unit (DU) was defined as the house (i.e. domestic sites) and structures included within the peridomestic area (e.g. kitchens, corrals, chicken coops); all 95 DU existing were georeferenced and numbered in the four communities TQ (10), JC (25), JG (28) and CA (32). The distances among the communities are between 3–7 km.

The capture of triatomines was carried out in two periods in 2014, January and October (8 months after chemical intervention) by a team composed by two technicians, searching inside houses (intradomicile and peridomestic structures), according to standardized procedures of the Pan American Health Organization [31, 32]. The triatomines were separated in plastic bottles by DUs and ecotopes, and subsequently reared in the laboratory for future analysis.

After the entomological evaluation in the four communities of municipality of Toro Toro, all infested DUs were sprayed with alphacypermethrin at nominal doses of 50 mg a.i./m^2^, using Hudson X-pert^TM^ manual sprayers, as indicated by the Pan American Health Organization protocol [33].

In parallel to the capture of domestic triatomines, wild populations were collected using traps described by Noireau et al. [34, 35] in ecotopes where the presence of wild triatomines was suspected, in parallel with the capture in the houses. The distance of the sylvatic ecotopes and the nearest houses was between 50–100 m for JC and JG, respectively. The number of traps varied depending on the available sampling area. A total of 30 traps (15 per evaluation) and 100 traps (50 per evaluation) were placed in Julo Chico and Julo Grande, respectively.

All insects collected were identified by using the taxonomic key of Lent & Wygodzinsky [3] and maintained under controlled conditions of temperature and humidity (25 °C ± 1 °C; 60% ± 10% RH).

### Chemicals

Deltamethrin technical grade (99.1%), obtained from Bayer CropScience (Brazil), was used for the bioassay, following the routine of “Monitoring Network for Triatominae Insecticide Resistance”, FIOCRUZ [36]. The acetone analytical grade used for dilutions was purchased from Merck, Germany. To test the susceptibility to insecticide, the triatomine colonies were founded after each phase of the work from a minimum of five insects [37] captured in the two phases of the work described in Table 1.

**Table 1:** Number of *T. infestans* captured in the domestic and sylvatic ecotopes, in four communities of Toro Toro, Potosi, Bolivia, before and after spraying (January and October 2014)

Community	Houses		Before spraying			After spraying		
	No. visited	No. positive	Intra (*n*)	Peri (*n*)	Wild (*n*)	Intra (*n*)	Peri (*n*)	Wild (*n*)
Taqó Taqó	10	1	0	48	0	0	3	0
Julo Chico	25	7	24	46	12	0	106	0
Julo Grande	28	6	31	10	11	57	27	5
Calahuta	32	5	3	123	0	0	26	0

*Abbreviations*: *Intra* intradomestic, *Peri* peridomestic, *n* number of individuals

### Bioassays

Although the chemical control in the municipality of Toro Toro is performed with alphacypermethrin, in the present work bioassays were realized using deltamethrin as reference insecticide, following the routine of the Laboratory of Reference in Triatomines and Epidemiology of Chagas Disease, FIOCRUZ, Belo Horizonte. Reference Center in Triatomine Resistance Studies for the Brazilian Ministry of Health and is recognized as a Collaborator Center for WHOPES.

The susceptible reference lineage (SRL) CIPEIN came from Centro de Investigaciones de Plagas e Insecticidas (CIPEIN) [38]. The baseline of susceptibility to deltamethrin of this lineage was determined by Gomez et al. [25] in Laboratory of Reference in Triatomines and Epidemiology of Chagas Disease, FIOCRUZ, Belo Horizonte.

Tests to determine insecticide susceptibility were conducted for each population; where a minimum of eight and a maximum thirteen doses by populations of active ingredient (a.i.), were applied ranging from 0.42 to 55 ng. For each insecticide dose, three replicates were performed with 10 instar F1 generation nymphs (five days, fasting, weight of 1.2 ± 0.2 mg). The topical application was through 0.2 μl of insecticide dilution in the dorsal abdomen, according to the procedures of the World Health Organization [39] and Pessoa et al. [40]. After treatment, mortality was assessed 72 h post-application and determined by the inability or lack of coordination of the nymphs to move from center to the edge of the filter paper (7 cm diameter). Signs of paralysis and lack of response to external stimuli were also considered. During and after the experiment, insects were kept under controlled conditions of temperature and relative humidity (25 °C ± 1 °C; 60% ± 10% RH).

### Diagnostic dose

The diagnostic doses (DD) applied was twice the minimum of the insecticide that causes 99% of mortality in the susceptible laboratory strain [20, 25, 41]. According to Gomez et al. [25] the LD99 to deltamethrin of the SRL was determined (5.50 ng a.i per insect) and the DD was estimated. According to Brown & Pal [42], mortality > 80% (DD) detects the presence of resistant individuals in a population. Values of RR_50_ > 5, according to the criteria established by PAHO [43], were considered as resistant to deltamethrin.

### Data analysis

The dose data/mortality was analyzed using the program PoloPlus version 2.0 [44]. The lethal doses required to kill 50% of treated individuals (LD_50_) was estimated and the resistance ratio (RR_50_), with their respective confidence intervals (95% CI).

## Results

In the four communities, 305 and 221 triatomines were captured before and after spraying, respectively. All captured triatomines were identified as *T. infestans*. Nineteen DUs were infested in the first phase and 11 in the second phase of the study (7 DUs were recurrent of the first phase). Two sylvatic foci were found, one in the communities of JC and another in JG; after eight months, only JG was positive in the second phase (Table 1).

Sixteen samples were obtained in the first capture, defined by the different domestic structures in the intra and peridomestic environments, and two in the sylvatic environments. All populations were resistant to deltamethrin (Table 2). The values of RR_50_ were 28.90 in the intradomicile to 54.7 for the peridomicile, and the wild population showed RR_50_ of 29.21–38.21. In the second capture of 13 populations of *T. infestans* from domestic and wild environments, all were resistant to deltamethrin (Table 3), with values RR_50_ of 21.91–40.67.

**Table 2:** Toxicological profile of *T. infestans* populations of domestic and sylvatic ecotopes, evaluated in the communities of Taqó Taqó, Julo Chico, Julo Grande and Calahuta, Potosi, Bolivia, before spraying with alphacypermethrin 50 mg a.i./m^2^ (January 2014)

CD	Ecotope (I, P, S)	Before spraying			
		LD_50_ (95% CI) (*n*)	Slope ± SE	RR_50_ (95% CI)	(*n*) DD%
CIPEIN (SRL)		0.47 (0.32–0.51) (210)	3.03 ± 0.37		
TQ 1	Goat corral (P)	12.78 (10.88–14.59) (216)	3.80 ± 0.44	31.37 (25.08–39.25)	(30)14.8
JC 2	Kitchen wall (P)	12.33 (9.9–14.6) (270)	2.57 ± 0.32	30.26 (23.46–39.03)	(30) 23.3
JC 3(a)	Deposit (P)	11.43 (10.01–12.81) (270)	3.57 ± 0.39	28.06 (22.75–34.61)	(30) 13.3
JC 3(b)	Goat corral and chicken coop (P)^a^	22.29 (20.04–24.59) (300)	4.06 ± 0.40	54.70 (44.83–66.73)	(30) 0
JC 4	Goat corral (P)	12.41 (8.37–16.98) (240)	2.73 ± 0.32	30.47 (24.06–38.60)	(30) 23.3
JC 7	Chicken coop (P)	13.06 (10.734–15.26) (240)	2.74 ± 0.35	32.06 (25.15–40.87)	(30) 23.3
JC Sil	Rocky outcrop (S)	15.57 (14.09–17.07) (300)	4.54 ± 0.43	38.21 (31.42–46.47)	(30) 3.3
JG 1	Goat corral (P)	10.45 (9.11–11.84) (300)	3.09 ± 0.31	25.66 (20.69–31.81)	(30) 16.7
JG 3	Kitchen roof (P)	18.85 (16.79–20.84) (240)	4.02 ± 0.53	46.27 (37.85–56.57)	(30) 0
JG 4(a)	Bedroom (I)	14.72 (13.28–16.24) (300)	3.98 ± 0.38	36.12 (29.63–44.04)	(30) 0
JG 6	Bedroom (I)	11.77 (10.55–12.99) (240)	4.76 ± 0.49	28.90 (23.67–35.28)	(30) 6.6
JG Sil	Rock cliff (S)	11.90 (10.55–13.25) (270)	4.14 ±0.41	29.21 (23.80–35.86)	(30) 6.6
CA 1	Living room wall (P)	13.27 (11.64–14.90) (390)	3.00 ± 0.27	32.57 (26.38–40.22)	(30) 6.6
CA 2	Living room wall (P)	22.28 (17.97–27.34) (270)	3.36 ± 0.38-	54.67 (44.42–67.29)	(30) 0
CA 5(a)	Goat corral (P)	15.63 (13.99–17.23) (300)	4.36 ± 0.43	38.35 (31.41–46.82)	(30) 3.3
CA 5(b)	Dog house (P)	15.99 (14.64–17.39) (240)	5.53 ±0.57	39.25 (32.43–47.50)	(30) 0

*Abbreviations*: *CD* code triatomine population, *SRL* susceptible reference lineage, *I* intradomestic, *P* peridomestic, *S* sylvatic, *n* number of individuals used, *LD*_*50*_ lethal dose that killed 50% of the population (ng.a.i./insect), *CI* confidence interval, *RR*_*50*_ resistance ratio, *DD* % mortality at the diagnostic dose

^a^Mixed structure of goat corral and chicken coop

**Table 3:** Toxicological profile of *T. infestans* populations of domestic and sylvatic ecotopes, evaluated in the communities of Julo Chico, Julo Grande and Calahuta, Potosi, Bolivia, after spraying with alphacypermethrin 50 mg a.i./m^2^ (October 2014)

CD	Ecotope (I, P, S)	After spraying			
		LD_50_ (95% CI) (*n*)	Slope ± SE	RR_50_ (95% CI)	(*n*) DD%
CIPEIN (SRL)		0.47 (0.32–0.51) (210)	3.03 ± 0.37		
JC 2	Kitchen wall (P)	12.11 (10.40–13.81) (270)	3.17 ± 0.34	29.72 (23.81–37.08)	(30) 6.7
JC 3(b)	Goat corral and chicken coop (P)^a^	14.72 (11.95–17.37) (240)	3.55 ± 0.42	36.13 (29.07–44.90)	(30) 13.3
JC 8	Goat corral (P)	11.10 (9.38–12.68) (270)	3.40 ± 0.39	27.26 (21.74–34.18)	(30) 10
JG 3	Kitchen roof (P)	10.47 (6.66–13.75) (180)	2.89 ± 0.44	25.70 (19.93–33.15)	(30) 30
JG 4(a)	Bedroom (I)	14.44 (10.72–17.94) (270)	2.51 ± 0.30	35.45 (27.66–45.43)	(30) 13.3
JG 4(b)	Tree with chickens (P)	12.71 (10.32–14.90) (270)	2.72 ± 0.33	31.19 (24.34–39.96)	(30) 13.3
JG 4(c)	Chicken coop (P)	10.22 (8.47–11.84) (180)	3.59 ± 0.48	25.10 (19.82–31.78)	(30) 16.7
JG 5	Bedroom wall (P)	11.03 (7.80–13.93) (210)	3.49 ± 0.43	27.08 (21.49–34.12)	(30) 23.3
JG 6	Bedroom (I)	11.34 (9.19–13.32) (210)	2.92 ± 0.40	27.84 (21.72–35.68)	(30) 13.3
JG Sil	Rock cliff (S)	16.57 (10.86–22.19) (270)	2.45 ± 0.32	40.67 (32.04–51.64)	(30) 20.0
CA 2	Living room wall (P)	11.34 (9.12–13.37) (210)	3.80 ± 0.39	27.83 (21.63–35.80)	(30) 23.3
CA 3	Goat corral (P)	8.92 (5.09–11.67) (180)	3.41 ± 0.54	21.91 (17.23–27.85)	(30) 33.3
CA 5(a)	Goat corral (P)	16.51 (12.55–19.97) (210)	5.88 ± 0.72	40.52 (33.41–49.15)	(30) 3.3

*Abbreviations*: *CD* code triatomine population, *SRL* susceptible reference lineage, *I* intradomestic, *P* peridomestic, *S* sylvatic, *n* number of individuals used, *LD*_*50*_ lethal dose that killed 50% of the population (ng.a.i./insect), *CI* confidence interval, *RR*_*50*_ resistance ratio, *DD* % mortality of the diagnostic dose

^a^Mixed structure of goat corral and chicken coop

All populations evaluated before and after spraying showed mortality lower than 33% for the diagnostic dose (DD). Comparing before and after spraying at the microgeographical level, the toxic response was different for triatomines within the same DU in the different communities. Of the seven DU positives (before and after fumigation), four had similar values of (JC2, JG4a, JG6, CA5a) and in three DUs, lower resistance after spraying (JC3b, JG3, CA2) was observed (Tables 2 and 3).

## Discussion

This study shows the high resistance to deltamethrin by domestic and wild *T. infestans* populations in four communities of the municipality of Toro Toro, Bolivia, before and eight months after spraying. Of particular importance are the results at the microgeographical level, demonstrating different resistance values in structures of the same DU. The calculation of RR_50_ is an important and frequent indicator of triatomine resistance to insecticides besides the diagnostic dose DD [17, 20, 25, 45], as it provides information about the mortality of a population in contact with different doses of insecticide, and can be employed to detect the presence of resistant individuals in a population [42]. How these indicators vary was evidenced in our measurements in an artificial environment (a mixed structure used as goat corral and chicken coop) before and after spraying: housing 3(b) of Julo Chico had a RR_50_ of 54.7 and RR_50_ of 36.13, contrary to what was observed in environments intradomicile of the house 4(a) of Julo Grande (RR_50_ of 36.12 to RR_50_ of 35.45). The variation in the wild environment of Julo Chico (RR_50_ of 38.21) and Julo Grande (RR_50_ of 29.21 to RR_50_ of 40.67) was also variable.

There was also variation in the values observed inside the same house (house 3 of Julo Chico), among the insects captured in different ecotypes and in the same period: deposit with RR_50_ of 28.6 and goat corral and chicken coop with RR_50_ of 54.7.

These different resistance profiles in different DUs from the same communities show the different geographical structuring of the resistance phenotype among the communities and ecotypes within sites at the microgeographical level. Already among the wild populations these showed profiles of high insecticide resistance. Thus, the persistence of these residual foci is probably related to the low efficiency of the insecticide applied in these communities. According to what was observed in areas of the Gran Chaco, both environmental factors [22, 46, 47] and existing surface characteristics would have different effects on the applied insecticide, conditioning or limiting its insecticidal action. In this situation, the insects would be exposed to sublethal doses, selecting resistant insects [13, 48, 49].

On the other hand, studies of *T. infestans* wild populations indicate that diverse regions of Bolivia [25, 26, 49] present different resistance profiles to insecticides. Wild populations from the Inter-Andean Valleys (Andean region) and the Gran Chaco (non-Andean region) resulted in resistance to deltamethrin with values ranging between 6.8 and 11.9 [26, 48]. Fipronil resistance varies between 5.5–45.6 [26], although this insecticide has never been used to triatomine control. In parallel, in these regions other wild *T. infestans* populations were reported susceptible [25].

Our results of wild *T. infestans* population showed high rates of resistance, ranging between 29.21–40.67. According to our observations and information from local authorities, the sylvatic area studied has never presented anthropogenic changes (such as agriculture or pasture), due its topography with predominance of rocky hills. As described in other regions, it seems that this deltamethrin resistance could be autochthonous, and the origin of the resistant domiciliary insects. The other way, from the houses to the sylvatic environment, had never been described for *T. infestans*. Nonetheless, it must be investigated using molecular markers.

Conversely, the presence of different profiles of residual foci of *T. infestans* in Bolivia indicates the occurrence of independent evolutionary processes of resistance to insecticides in the different regions. The high genetic variability described for *T. infestans* by Torres-Perez et al. [50] and Panzera et al. [51] justifies Bolivia as the center of origin and dispersion of the species, which could explain the reason why natural insecticides resistance have risen in this country [52].

## Conclusions

The results of the present study demonstrated high resistance to deltamethrin of domestic and sylvatic *T. infestans* of Toro Toro, Potosi, Bolivia, varying at the macro- and microgeographical levels; this is more complex when considering the importance of many factors that act over the biological performance of a population, e. g. reproductive capacity, viable offspring, obtaining blood capacity, dispersion capacity, and others, that should be investigated.

## Abbreviations

a.i.: active ingredient
RH: relative humidity
